# Stability limits of tin-based electrocatalyst supports

**DOI:** 10.1038/s41598-017-04079-9

**Published:** 2017-07-04

**Authors:** Simon Geiger, Olga Kasian, Andrea M. Mingers, Karl J. J. Mayrhofer, Serhiy Cherevko

**Affiliations:** 1 Department of Interface Chemistry and Surface Engineering, Max-Planck-Institut für Eisenforschung GmbH, 40237 Düsseldorf, Germany; 2 Helmholtz-Institute Erlangen-Nürnberg for Renewable Energy (IEK-11), Forschungszentrum Jülich GmbH, Erlangen, 91058 Germany; 3 Department of Chemical and Biological Engineering, Friedrich-Alexander-Universität Erlangen-Nürnberg, 91058 Erlangen, Germany

## Abstract

Tin-based oxides are attractive catalyst support materials considered for application in fuel cells and electrolysers. If properly doped, these oxides are relatively good conductors, assuring that ohmic drop in real applications is minimal. Corrosion of dopants, however, will lead to severe performance deterioration. The present work aims to investigate the potential dependent dissolution rates of indium tin oxide (ITO), fluorine doped tin oxide (FTO) and antimony doped tin oxide (ATO) in the broad potential window ranging from −0.6 to 3.2 V_RHE_ in 0.1 M H_2_SO_4_ electrolyte. It is shown that in the cathodic part of the studied potential window all oxides dissolve during the electrochemical reduction of the oxide – *cathodic dissolution*. In case an oxidation potential is applied to the reduced electrode, metal oxidation is accompanied with additional dissolution – *anodic dissolution*. Additional dissolution is observed during the oxygen evolution reaction. FTO withstands anodic conditions best, while little and strong dissolution is observed for ATO and ITO, respectively. In discussion of possible corrosion mechanisms, obtained dissolution onset potentials are correlated with existing thermodynamic data.

## Introduction

Apart from their widespread application as transparent conducting materials in optoelectronics, photovoltaic, etc., recently tin-based oxides have also attracted attention as alternative support materials in the field of electrochemistry. Typically discussed advantages of using such oxides are relatively good stability and conductivity^1–4^. Additionally, tin-based oxides are suggested to enhance stability and activity of the catalyst due to the so called strong catalyst-support interaction^2, 5–7^.

Three tin-based conductive oxide supports, extensively used in electrochemistry, are: indium tin oxide (ITO), antimony doped tin oxide (ATO), and fluorine doped tin oxide (FTO). Literature on the lab-scale application of these oxides is extensive, just a short overview is given here. ITO is considered as a support for Pt, catalysing oxygen reduction reaction (ORR) in anion exchange membrane fuel cells^2^, oxidation of formaldehyde^8^ or methanol^9, 10^, amperometric sensors^11^; as well as support for IrO_2_^12^, molecular iridium^13^, and silver oxide^14^ for the oxygen evolution reaction (OER). ATO is used as alternative support on the cathode of proton exchange membrane (PEM) fuel cells^3, 15, 16^ as well as for the OER in PEM electrolysers^5, 7, 17^ making it a promising material for regenerative fuel cells^18^. FTO is applied in solar cells, waste water treatment, and many other fields of electrochemistry^1, 13, 19–24^.

Although these oxides are generally considered more stable than carbon (state-of-the-art support material in fuel cells and also considered for applications in water electrolysis), in harsh electrochemical conditions they also degrade. Hence, in order to assess its potential application in electrochemical devices, e.g. electrolysers or fuel cells, detailed understanding of the degradation processes is essential. As a first step, extensive knowledge of carbon supports degradation in fuel cells, generated in the last decades, can be used. Similarly to carbon, corrosion of tin-based oxide support may lead to detachment of the catalyst. There are, however, processes which are inherent to oxide supports. Most crucial is dissolution. Since conductivity of tin-based oxides depends on the concentration of dopants, even a small decrease in their amount may lead to a significant increase in ohmic losses. The latter will result in a decrease in energy efficiency. Moreover, dissolution products of both tin and dopant metal may end up in the membrane or on the counter electrode, deteriorating their performance.

In the light of the above, it is not surprising that degradation of ITO, ATO, and FTO was numerously addressed in literature. Thus, ITO and FTO were studied in a comprehensive overview of substrate materials for electrochemical applications, in which a broader stability of FTO was claimed^25^. Most of the results on stability originate from pre- and post-measurements of the oxides resistivity, capacitance, optical properties, and surface composition^1, 3, 15, 16, 26–35^. Only in a few works dissolution was actually estimated, e.g. by quartz crystal microbalance^36–38^ or using radiochemical methods^39^. In these latter studies, however, neutral and/or chloride containing electrolytes, important for photovoltaic applications but not PEM fuel cells and electrolysers, were used. Moreover, only the total but not partial dissolution rates of the respective elements were shown. The current work aims to fill this gap in the existing literature. It presents a detailed study on time- and potential-resolved dissolution of Sn, In, and Sb from FTO, ITO, and ATO in acidic electrolyte in a broad potential window covering the important regions of hydrogen and oxygen evolution. Quantification of the trace amounts of dissolved material and estimation of experimental onset potentials was made possible by using an inductively coupled plasma mass spectrometer (ICP-MS), which was hyphenated to an electrochemical scanning flow cell (SFC). The collected data were used in the description of the tentative dissolution mechanisms based on the available thermodynamics data^40^ and in the discussion of the kinetics effects.

## Results and Discussion

**Figure 1:**
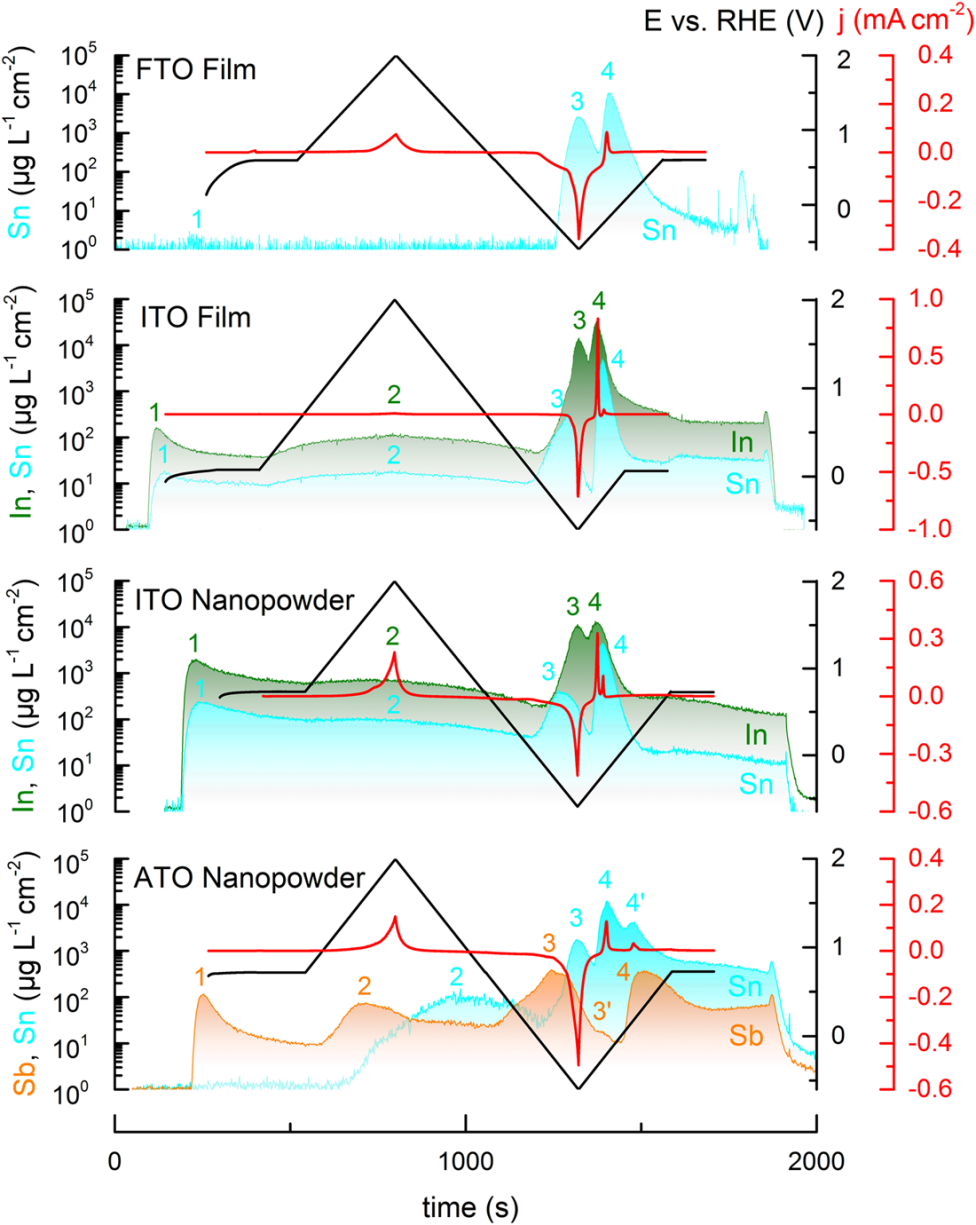
Corrosion of FTO, ITO and ATO studied by tracking the metal concentration in the electrolyte at open circuit potential and during a slow scan (5 mV s^−1^) to anodic and cathodic region in 0.1 M H_2_SO_4_ purged with argon.

In order to get a first look on the potential dependence of ITO, ATO, and FTO dissolution a potential program shown as black curve in Fig. 1 was applied to each of the studied electrodes. It consists of a slow potential sweep (5 mV s^−1^) from the open circuit potential (OCP) into the anodic region up to 2.0 V_RHE_; a cathodic sweep down to −0.6 V_RHE_; and the final sweep from the cathodic vertex potential back to the initial OCP. The current response is coloured in red and the resulting metal concentration in the electrolyte, monitored by SFC-ICP-MS, is depicted in three other colours in the same figure. A standard plot of current vs. potential in Fig. S5 facilitates the interpretation of the electrochemical data. The most significant dissolution is observed during the reduction of the oxides (peak 3) and during the re-oxidation of the formed metal in the following anodic sweep (peak 4). This phenomenon, which holds for all studied materials, can be nicely correlated with the corresponding cathodic and anodic currents. Besides these major processes some dissolution is also observed during the initial contact of the oxides with electrolyte at OCP (peak 1) and anodic polarization (peak 2).

Below we discuss separately dissolution taking places in the peaks 1, 2 and 3, 4. First, however, macroscopic reactions which can be responsible for the observed corrosion are summarized for pH 1 and 1 nM of the equilibrium concentration of the dissolved species in the electrolyte^40^. The reactions are split in three major dissolution processes:

a) Cathodic dissolution:

1 $$\begin{array}{ll}{{\rm{SnO}}}_{{\rm{2}}}+{{\rm{4H}}}^{+}+{{\rm{2e}}}^{-}\rightleftarrows {{\rm{Sn}}}^{{\rm{2}}+}+{{\rm{2H}}}_{{\rm{2}}}{\rm{O}} & {\rm{E}}=+0.07\,\,{{\rm{V}}}_{{\rm{SHE}}}\end{array}$$

2 $$\begin{array}{ll}{{\rm{In}}}_{{\rm{2}}}{{\rm{O}}}_{{\rm{3}}}+{{\rm{6H}}}^{+}+{{\rm{4e}}}^{-}\rightleftarrows {{\rm{2In}}}^{+}+{{\rm{3H}}}_{{\rm{2}}}{\rm{O}} & {\rm{E}}=-0.04\,{{\rm{V}}}_{{\rm{SHE}}}\end{array}$$

3 $$\begin{array}{ll}{{\rm{Sb}}}_{{\rm{2}}}{{\rm{O}}}_{{\rm{5}}}+{{\rm{6H}}}^{+}+4{{\rm{e}}}^{-}\rightleftarrows {{\rm{2SbO}}}^{+}+{{\rm{3H}}}_{{\rm{2}}}{\rm{O}} & {\rm{E}}=+0.76\,\,{{\rm{V}}}_{{\rm{SHE}}}\end{array}$$

b) Anodic dissolution:

4 $$\begin{array}{ll}{\rm{Sn}}\rightleftarrows {{\rm{Sn}}}^{2+}+{\rm{2}}{e}^{-} & {\rm{E}}=-0.40{{\rm{V}}}_{{\rm{SHE}}}\end{array}$$

5 $$\begin{array}{ll}{\rm{In}}\rightleftarrows {{\rm{In}}}^{3+}+{\rm{3}}{e}^{-} & {\rm{E}}=-0.52{{\rm{V}}}_{{\rm{SHE}}}\end{array}$$

6 $$\begin{array}{ll}{\rm{Sb}}+{{\rm{H}}}_{{\rm{2}}}{\rm{O}}\rightleftarrows {{\rm{SbO}}}^{+}+{{\rm{2H}}}^{+}+{{\rm{3e}}}^{-} & {\rm{E}}=-0.01{{\rm{V}}}_{{\rm{SHE}}}\end{array}$$

c) Chemical dissolution:

7 $$\begin{array}{ll}{{\rm{SnO}}}_{{\rm{2}}}+{{\rm{4H}}}^{+}\rightleftarrows {{\rm{Sn}}}^{{\rm{4}}+}+{{\rm{2H}}}_{{\rm{2}}}{\rm{O}} & [{{\rm{Sn}}}^{4+}]={10}^{-11}{\rm{M}}\end{array}$$

8 $$\begin{array}{ll}{{\rm{In}}}_{{\rm{2}}}{{\rm{O}}}_{{\rm{3}}}+{{\rm{6H}}}^{+}\rightleftarrows {{\rm{2In}}}^{{\rm{3}}+}+{{\rm{3H}}}_{{\rm{2}}}{\rm{O}} & [{{\rm{In}}}^{3+}]={10}^{+4}{\rm{M}}\end{array}$$

9 $$\begin{array}{ll}{{\rm{Sb}}}_{{\rm{2}}}{{\rm{O}}}_{{\rm{5}}}+{{\rm{2H}}}^{+}\rightleftarrows {{\rm{2SbO}}}_{{\rm{2}}}^{+}+{{\rm{H}}}_{{\rm{2}}}{\rm{O}} & [{{{\rm{SbO}}}_{2}}^{+}]={10}^{-6}{\rm{M}}\end{array}$$

10 $$\begin{array}{ll}{{\rm{Sb}}}_{{\rm{2}}}{{\rm{O}}}_{{\rm{5}}}+{{\rm{H}}}_{{\rm{2}}}{\rm{O}}\rightleftarrows {{\rm{2SbO}}}_{{\rm{3}}}^{-}+{{\rm{2H}}}^{+} & [{{{\rm{SbO}}}_{3}}^{-}]={10}^{-3}{\rm{M}}\end{array}$$

Dissolution during OCP (peak 1) can be explained by chemical dissolution of the oxides as indicated by equations 7–10 with the following tendency for dissolution taken from the equilibrium concentration of the dissolved ions: In_2_O_3_ ≫ Sb_2_O_5_ ≫ SnO_2_. Note, a direct comparison between ITO and ATO in the experiment is difficult since the portion of In_2_O_3_ and Sb_2_O_5_ differs (see Table S1). The dissolution signal of Sn (ITO) is significantly higher compared to Sn (FTO/ATO), which indicates destabilization of Sn in an In-Sn-oxide. The reason for this could be intensive dissolution of In, leaving behind prone to dissolution under-coordinated Sn atoms.

Dissolution during anodic polarization (peak 2) cannot be easily assigned to any of the electrochemical reactions presented above^40^. Still, the dissolution increase with potential, presented in Fig. 1, is a sign that the dissolution is rather an electrochemical than chemical process. Only for Sb, dissolution can be assigned using information on thermodynamics of the oxides^40^. Surface states of Sb are expected to be mostly Sb^III^ species while bulk material consists of Sb^V^ ^16, 41^. Therefore increased detection of Sb towards anodic potentials could be explained by:

11 $$\begin{array}{ll}{{\rm{Sb}}}_{{\rm{2}}}{{\rm{O}}}_{{\rm{3}}}+{{\rm{3H}}}_{{\rm{2}}}{\rm{O}}\rightleftarrows {{\rm{2SbO}}}_{{\rm{3}}}^{-}+{{\rm{6H}}}^{+}+{{\rm{4e}}}^{-} & {\rm{E}}=+0.47{{\rm{V}}}_{{\rm{SHE}}}\end{array}$$

**Figure 2:**
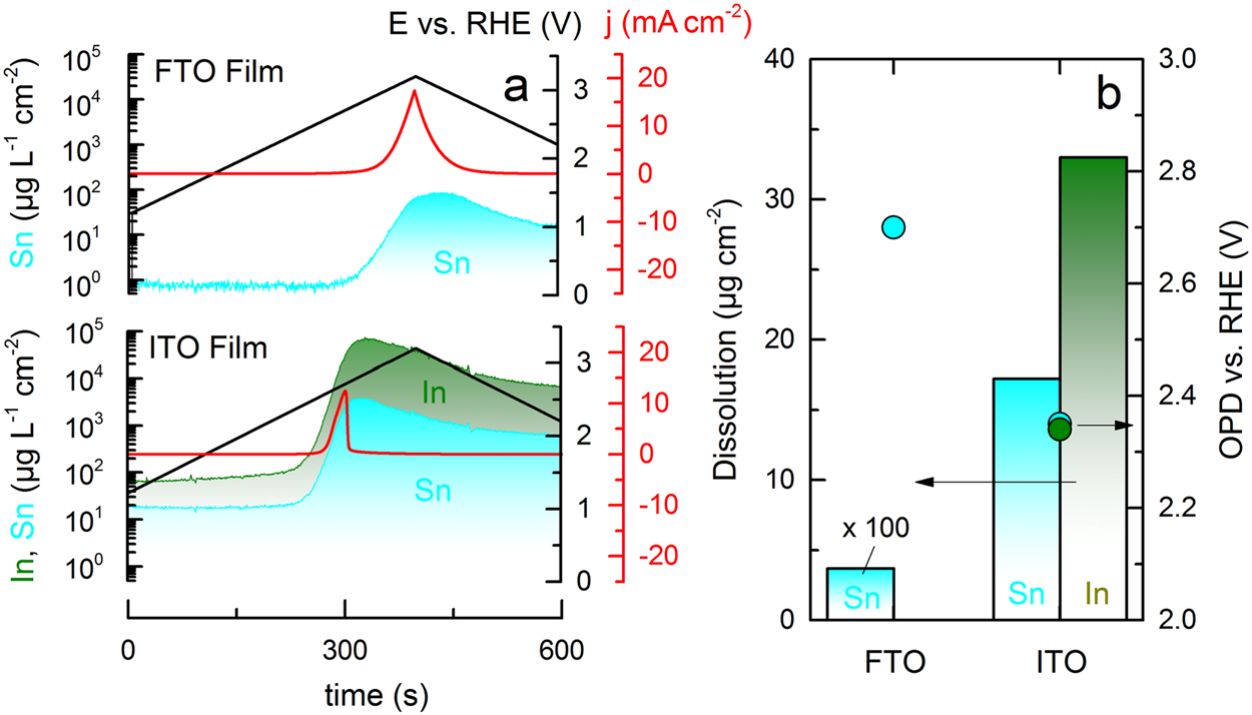
(**a**) Corrosion of FTO and ITO films during a slow scan (5 mV s^−1^) to 3.2 V_RHE_ in 0.1 M H_2_SO_4_ purged with argon. (**b**) Comparison of the integrated dissolution during OER normalized by the portion of the respective element in the material (bars) and onset potential of dissolution, OPD (bullets).

To investigate dissolution of ITO and FTO during anodic polarization further, the anodic limit was extended to 3.2 V_RHE_. Corresponding potential program, current and dissolution signal are presented in Fig. 2, using the same colour code as in Fig. 1. Respective cyclic voltammograms are presented in Fig. S6. The increase in potential causes a steep raise in dissolution of ITO. Additionally, stable during the excursion to 2 V_RHE_, FTO starts to dissolve at the higher potentials. One can also see that the onset of dissolution coincides with the onset of the OER, clearly indicating a correlation of both processes. As the highest oxidation state is reached for the respective metal cations, oxidation of O^–2^ to molecular oxygen which is accompanied by metal dissolution can be suggested for the explanation of this effect^39, 42^:

12 $${{\rm{In}}}_{{\rm{2}}}{{\rm{O}}}_{{\rm{3}}}\to {{\rm{2In}}}^{{\rm{3}}+}+{\rm{1}}{{\rm{.5O}}}_{{\rm{2}}}+{{\rm{6e}}}^{-}$$

13 $${{\rm{SnO}}}_{{\rm{2}}}\to {{\rm{Sn}}}^{{\rm{4}}+}+{{\rm{O}}}_{{\rm{2}}}+{{\rm{4e}}}^{-}$$

14 $${{\rm{Sb}}}_{{\rm{2}}}{{\rm{O}}}_{{\rm{5}}}\to {{\rm{2Sb}}}^{{\rm{5}}+}+{\rm{2}}{{\rm{.5O}}}_{{\rm{2}}}+{{\rm{10e}}}^{-}$$

In order to obtain information on the amounts of dissolved material, the dissolution peaks from Fig. 2a were integrated. Moreover, potentials at which dissolution signal starts to deviate from the blank were indicated (onset potential of dissolution – OPD). Corresponding data is presented in Fig. 2b. Comparing dissolution of Sn from ITO and FTO, it is clear that the former is less stable – the onset of Sn dissolution is lower and the dissolution amount is more than two orders of magnitude higher for ITO. Indium dissolves even more, 30 µg cm^−2^ correspond to the loss of ca. 45 nm in the film thickness, which is approximately one third of the overall thickness. The amount of dissolved Sn corresponds to ca. 26 nm, adverting an enrichment of SnO_2_ during OER, which is in line with findings of Kraft *et al*.^39^. This might lead to decrease in conductivity explaining the drop in current at 2.7 V_RHE_. For ITO, assuming that Equations 12 and 13 are operative, 67% of the total current is originating from In and Sn dissolution, while the remaining 33% can be assigned to OER current. In the case of FTO, only 0.013% of the total current arises from dissolution of Sn. Note, in addition to equations 12–14 other dissolution processes cannot be excluded.

**Figure 3:**
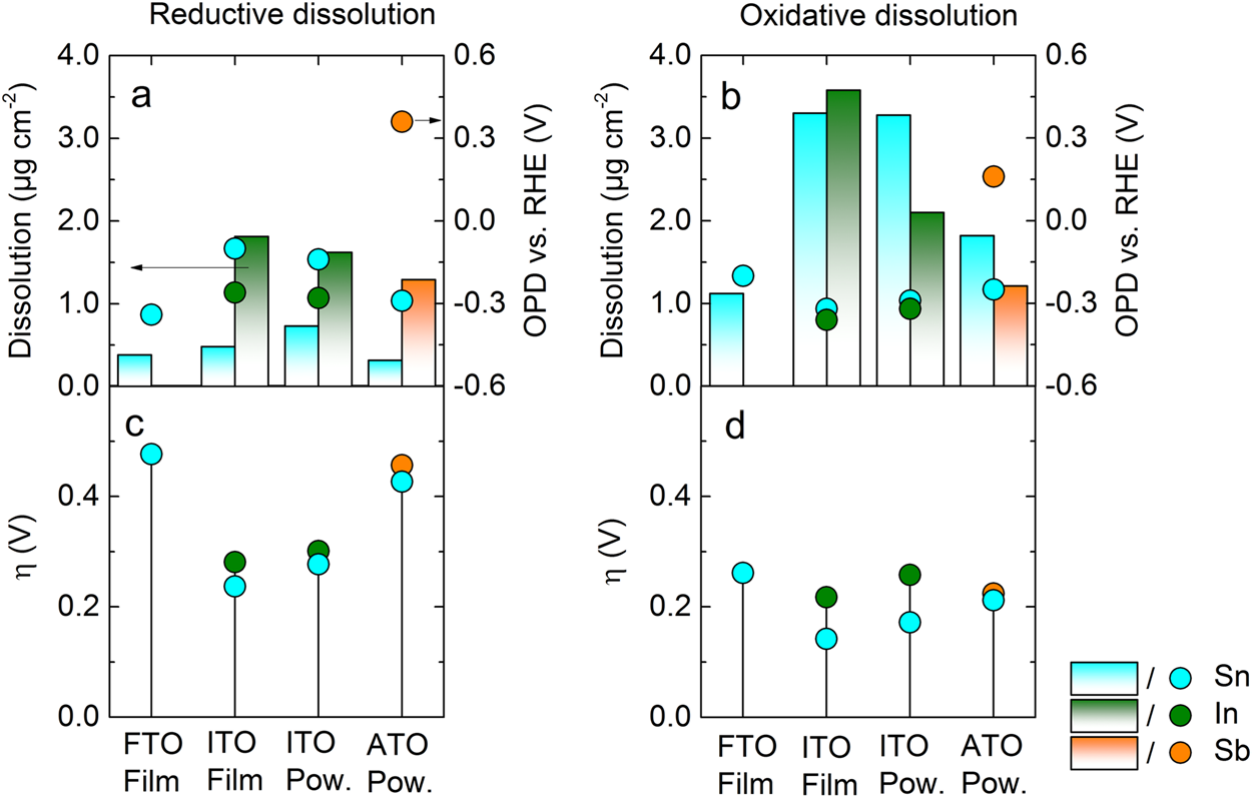
(**a**,**b**) Comparison of the integrated dissolution during reduction and reoxidation shown in bars. Values are normalized by the portion of the respective element in the material. Related onset potential of dissolution (OPD) is shown in bullets on the right axis. (**c,d**) Overpotential for the reductive and oxidative dissolution calculated from the difference between OPD and equilibrium potential at 1 nM (see equations 1–6).

Dissolution during reduction and reoxidation (peaks 3, 4) can be discussed on the basis of equations 1–3 and 4–6, respectively. Figure 3 summarizes the amounts of dissolved material in the corresponding peaks and the anodic and cathodic OPDs. Both parameters were extracted from Fig. 1. For FTO and ATO, the cathodic/reductive OPD deviates from the thermodynamically predicted values of about 400 mV, while for both film and powder ITO this value is about 200 mV (Fig. 3c). It can be suggested that the corresponding oxides in FTO and ATO are kinetically stabilized compared to ITO. It should be noted that the experimental values for OPD and dissolution overpotential (η), shown in Fig. 3c+d, depend on the time scale of experiment and the detection limit of the system (equilibrium concentration close to the electrode is estimated to be on the order of 1 nM) and therefore cannot be used as absolute values; however, comparison between materials is still possible. Hence, a lowered kinetic hindrance for the reductive dissolution of SnO_2_ observed in the case of ITO indicates a lower stability of Sn in the oxide with In. A similar trend is seen for anodic/oxidative OPD. Values for η are lower for the anodic dissolution of Sn in ITO (see Fig. 3d).

In terms of dissolved amounts, both In_2_O_3_ and Sb_2_O_3_ dissolve more during reduction compared to SnO_2_ (Fig. 3a). For all materials higher dissolution was observed during reoxidation of the formed metals (Fig. 3b). Especially dissolution of Sn is enhanced, which indicates a lower stability of Sn in an In-Sn alloy. This can be explained by In dissolution triggered destabilization of Sn, since the OPD for In is lower (see Fig. 3b). In other words, started at lower potentials, In dissolution results in formation of high surface area destabilized Sn-rich phases. Similar destabilization is also observed for ATO, clearly seen from Fig. 1, where the onsets of Sn and Sb dissolution in peaks 3 and 4 are well resolved. Here, an additional Sb dissolution peak 3´ appears in the region of anodic Sn dissolution (peak 4). On the other hand, dissolution of Sb triggers Sn depassivation (peak 4′).

Comparing total and dissolution current from Fig. 1, a dissolution efficiency was obtained for each element. For cathodic dissolution, most of the charge is used for reduction of oxides to metals and hydrogen evolution; except for In_2_O_3_, where the dissolution current counts as ~30% of the total current. On the other hand, the current in the oxidative branch is assumed to be predominantly due to dissolution. Corresponding data is summarized in Table 1. During oxidation the ratio of dissolution current to total current was found to exceed 100%, which might be caused by chemical dissolution of monoxides formed by partial reduction of the corresponding dioxides in the preceding reductive scan. Alternatively, electrochemical dissolution of species with other than the used charge number (including zero for neutral species/clusters) can be suggested.

**Table 1:** Dissolution charge (mC cm^−2^) during reduction and oxidation in relation to total charge.

		Reduction		Oxidation				Reduction		Oxidation	
FTO (Film)	Sn	0.62 (11)	5.6%	1.81 (1.46)	125%	ATO (Powder)	Sn	0.23 (15.1)	1.5%	2.25 (1.50)	148%
	F	—		—			Sb	0.33 (15.1)	2.2%	0.35 (0.37)	95%
ITO (Film)	Sn	0.08 (10)	0.8%	0.54 (0.45)	120%	ITO (Powder)	Sn	0.14 (12.5)	1.2%	0.53 (0.51)	103%
	In	2.7 (10)	27%	8.12 (4.4)	185%		In	3.63 (12.5)	29%	3.78 (3.35)	118%

Dissolution charge (calculated based on the amount of ions measured with ICP-MS) is presented for each element. Total charge (extracted from electrochemical data) is shown in brackets next to the dissolution charge. The used charge numbers are +1, +2, and +3 for Sb, Sn, and In, respectively. Ratio of dissolution charge and total charge is given in percentage.

### Consequences of dissolution

The last part of the manuscript is devoted to possible consequences of dissolution for the application of the oxides in electrochemical energy conversion devices. ITO is dissolving rapidly if the potential exceeds the limits of −0.1 V_RHE_ > E > 2.35 V_RHE_. In between these limits dissolution is lower but still significant. A simple calculation, with assumption that the dissolution rate is constant, reveals that a complete dissolution of a 10 nm film can be reached in just 12 h during OCP. Hence, ITO cannot be recommended for any application involving long stays at OCP in acidic electrolytes. Especially critical is the use of ITO as the catalyst support for hydrogen evolution reaction or electrochemical deposition of base metals.

For ATO, Sb and Sn oxides are relatively stable within 0.36 V_RHE_ < E < 1.1 V_RHE_ and −0.29 V_RHE_ < E < 1.45 V_RHE_, respectively. Hence, exceeding the stability window of Sb but not Sn, a SnO_2_ rich layer can be formed on the surface. This was also recently shown by Cognard *et al*. ^3^ who used X-ray photoelectron spectroscopy for analysis of the degraded ATO films in an accelerated stress test (1.0–1.5 V_RHE_). Consequences of such surface segregation of SnO_2_ should not be underestimated as a core shell structure limits the exchange of electrons^3^. Based on the initial dissolution during 5 min OCP, formation of a 0.2 nm thick SnO_2_ film due to preferential dissolution of Sb was estimated. Fabbri *et al*.^16^ also reported on the decrease in Sb amount after potential cycling (0.05–1.6 V_RHE_). According to the authors, however, reduction of calcination time and formation of films with a homogenous distribution of Sb lead to formation of a more stable Sb phase. Therefore, synthesis procedure and quality of the resulting films are essential parameters for stability of ATO.

The stability window of FTO ranges from −0.34 V_RHE_ < E < 2.7 V_RHE_ with no indication for any measurable dissolution in between these limits. In contrast to the other studied materials, doping is achieved by replacing oxygen with fluorine atoms instead of cation exchange. In this way less stable oxides are avoided, improving the overall stability of the material. Nevertheless, electrodeposition at very low potentials as well as CO_2_ reduction^35^ will lead to dissolution of Sn from FTO. Moreover, in chloride containing electrolytes stability of FTO can be an issue^37, 38^. Based on our study in ultrapure 0.1 M H_2_SO_4_, FTO shows best stability and is therefore considered as an appropriate candidate for many applications in electrochemistry. At the same time, however, FTO has the poorest intrinsic conductivity, which means that even a small decrease in performance can be detrimental. Further works should be dedicated to the effect of possible F-leaching and related influence on conductivity.

## Conclusions

This work provides a detailed investigation of the corrosion of tin-based oxides in acidic electrolyte in the absence of chlorides. All studied oxides, viz. ITO, ATO, and FTO, were unstable at high cathodic and anodic potentials. In terms of relative stability FTO was the most stable followed by ATO. ITO showed poor stability in the whole studied potential region. Dissolution during reduction is most critical for all studied materials questioning the application of these oxides in the cathodic range of potential. This process is assigned to the incomplete reduction of the oxide during the cathodic dissolution. As explanation for the anodic dissolution, surface disturbance due to the oxide lattice decomposition and oxygen evolution reaction was suggested. Based on the stability data, FTO can be considered as the best support.

## Methods

Electrochemical tests were performed in argon purged 0.1 M H_2_SO_4_ using a scanning flow cell (SFC) connected to an inductively coupled plasma mass spectrometer (ICP-MS)^43, 44^. A sketch of the setup is provided in the supporting information (Fig. S1). A graphite rod and an Ag/AgCl (Metrohm, Germany) electrode were used as counter and reference electrodes, respectively. The electrolyte was prepared by dilution of concentrated acid (Suprapur^®^ 96% H_2_SO_4_, Merck, Germany) in ultrapure water (PureLab Plus system, Elga, 18 MΩ cm, TOC < 3 ppb). Flow rate through the cell was 180 µL min^−1^. Steady performance of the ICP-MS (NexION 300X, Perkin Elmer) was ensured by addition of internal standard solution (^103^Rh, ^130^Te) downstream to the flow cell and daily calibration. Materials used as working electrodes are summarized in Table S1. Films and nanopowders of the oxides (ITO, FTO, ATO) were purchased from Sigma Aldrich. As ATO is just available as powder, for the sake of comparison, ITO was as well studied as powder. Before using the working electrodes, the films were rinsed with ethanol and ultrapure water. Nanopowders were supported by a glassy carbon plate. For preparation of an ink, 4 mg of the nanopowders were suspended in 5 ml of 0.001 M NaOH with addition of 20 µL of Nafion-solution (5 w% Sigma Aldrich). After ultrasonic treatment 0.5 µL of the suspension were dropcasted on the glassy carbon plate and let to dry. The dried spots (Ø ~ 1 mm) were rinsed with water and located with the help of a vertical camera attached to the SFC. The measurements on these electrodes were done by placing the spot in the centre of the SFC’s opening (Ø = 2 mm)^45^. Current and dissolution were normalized to the electrochemical surface area (ECSA). The geometric surface area was used for films as the roughness factor for sputtered films is assumed to be low. A rough estimation of the ECSA of ATO and ITO powders was done by measuring their capacitance and normalizing the obtained values to the capacitance of the films^46, 47^. For the latter, the measured value for ITO was 10 µF cm^−2^ and the estimated value for ATO was 50 µF cm^−2^, in accordance with literature. Also for FTO film measured capacitance was 50 µF cm^−2^. A more detailed description can be found in the SI (Fig. S7–S9 and related text).

## Electronic supplementary material

Supplementary Information
