# Supplementary material for: Stability limits of tin-based electrocatalyst supports
Source: Sci Rep. 2017 Jul 4;7:4595. doi: 10.1038/s41598-017-04079-9 (PMC5496880; doi:10.1038/s41598-017-04079-9)
Supplement: Supplementary file 1 — Supplementary Information [file 41598_2017_4079_MOESM1_ESM.pdf]

Supporting information:

Stability limits of tin-based electrocatalyst supports

Simon Geiger[a,*], Olga Kasian[a] , Andrea M. Mingers[a],

Karl J. J. Mayrhofer[a,b,c], Serhiy Cherevko[a,b,*]

[a] *Department of Interface Chemistry and Surface Engineering, Max-Planck-Institut für Eisenforschung GmbH, 40237 Düsseldorf, Germany*

[b] *Helmholtz-Institute Erlangen-Nürnberg for Renewable Energy (IEK-11), Forschungszentrum Jülich, 91058 Erlangen, Germany*

[c] *Department of Chemical and Biological Engineering, Friedrich-Alexander-Universität Erlangen-Nürnberg, 91058 Erlangen, Germany*

[*]*Corresponding authors: geiger@mpie.de, cherevko@mpie.de*

Table S1. Overview of the studied materials

Material$^{\Delta}$	Shape$^{\Delta}$	Composition$^{\Delta}$ (w%)	Purity$^{\Delta}$	BET surface$^{\Delta}$	ECSA†
**FTO** Fluorine doped tin oxide	Coated glass slide	SnO_2 (n.a.), F (n.a.)	n.a.	-	0.01 cm²
**ITO** Indium tin oxide	Coated glass slide (thickness 120 - 160 nm)	In_2O_3 (90%), SnO_2 (10%)	n.a.	-	0.01 cm²
**ITO** Indium tin oxide	Nanopowder (< 50 nm particle size)	In_2O_3 (90%), SnO_2 (10%)	n.a.	27 m²/g	0.0091 cm²
**ATO** Antimony doped tin oxide	Nanopowder (< 50 nm particle size)	Sb_2O_5 (7 - 11%), SnO_2 (89 - 93%)	≥ 99.5% trace metals basis	47 m²/g	0.032 cm²

$^{\Delta}$Provided by supplier †Determined in this work

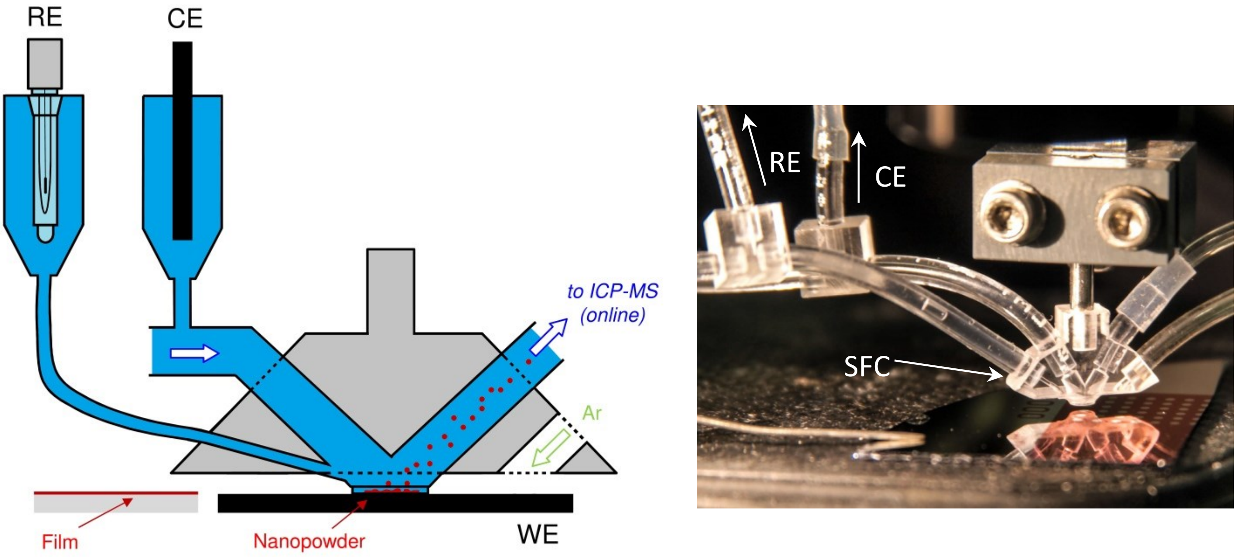

Figure S1. Schematic drawing and image of the Scanning Flow Cell (SFC).

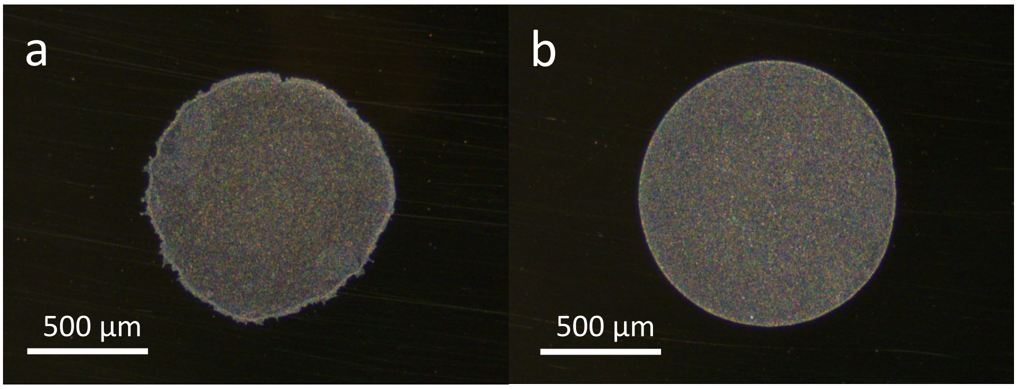

Figure S2. Image of the dropcasted nanopowders. **a** ATO, **b** ITO.

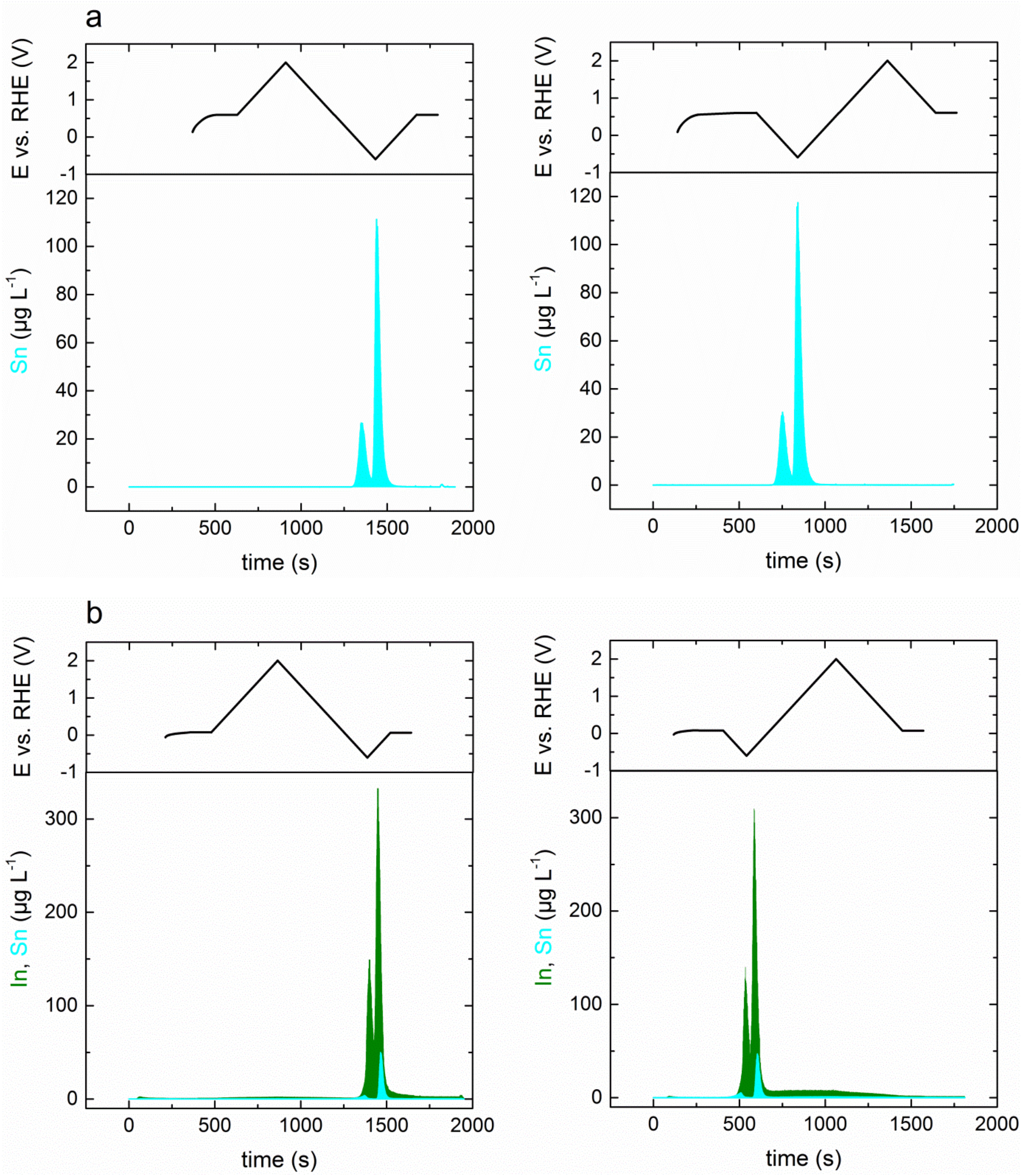

Figure S3. Corrosion of **a** FTO and **b** ITO films with slightly different measurement protocols to show reproducibility of the measurements and independence of the way the protocol is carried out.

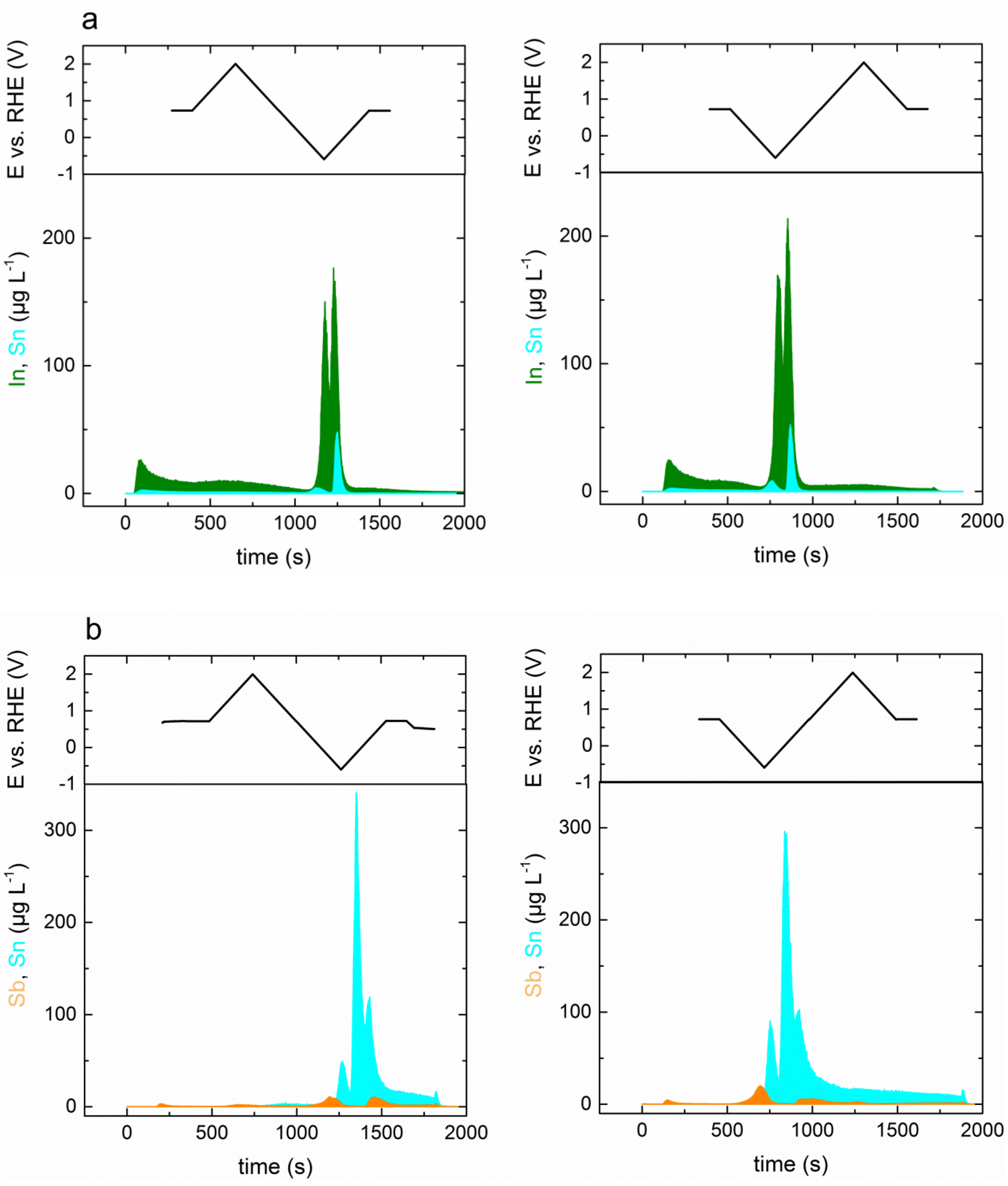

Figure S4. Corrosion of **a** ITO and **b** ATO nanopowders with two different measurement protocols to show reproducibility of the measurements and independence of the way the protocol is carried out.

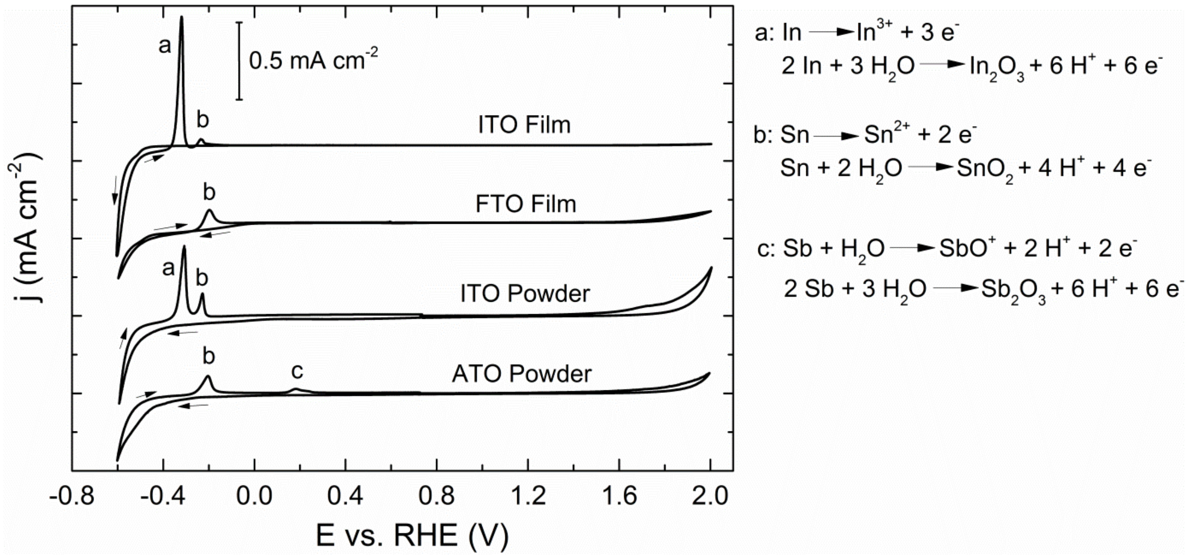

Figure S5. Cyclic voltammograms recorded at 5 mV s^{-1} in 0.1 M H_2SO_4. After reducing the oxides during a scan to – 0.6 V_{RHE}, oxidative peaks appear in the reverse scan, highlighted as a, b, and c. We tried to separate the dissolution pathway from oxidation pathway by comparing total charge with total amount of dissolved ions (see discussion in the main text and Table 1).

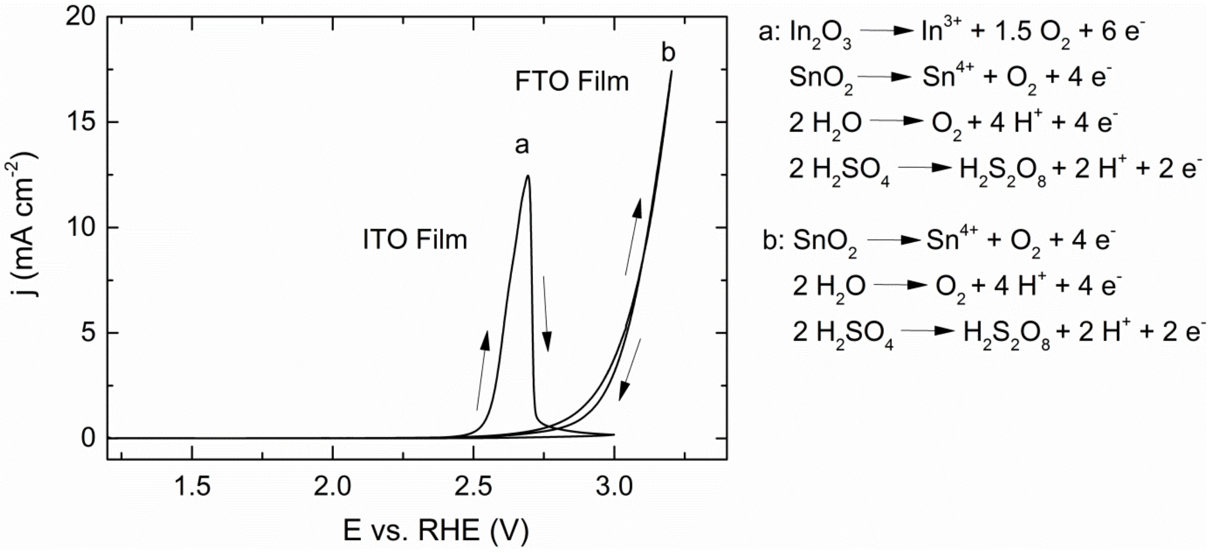

Figure S6. Cyclic voltammograms recorded at 5 mV s^{-1} in 0.1 M H_2SO_4. Possible reaction pathways are highlighted for ITO and FTO, respectively.

Estimation of the electrochemical surface area (ECSA):

First, the double layer capacitance was estimated on flat FTO, ITO and GC samples with known geometric surface area using cyclic voltammetry (see first raw in Fig. S1). The method is described in detail elsewhere[1,2]. Arrows in Fig. S1 indicate the potential at which capacitance current was estimated. These potentials are chosen with a view of having the minimal faradaic current. The second column in Fig. S1 presents scan rate (υ) dependence of the half the sum of anodic and cathodic current (j), from which double layer capacitance is obtained as a slope of the j vs. υ line. The obtained values for the double layer capacitance were around 50 μF cm^{-2} for FTO and GC and 10 μF cm^{-2} for ITO. These values are used below for estimation of the electrochemical surface area (ECSA) of ITO and ATO powders.

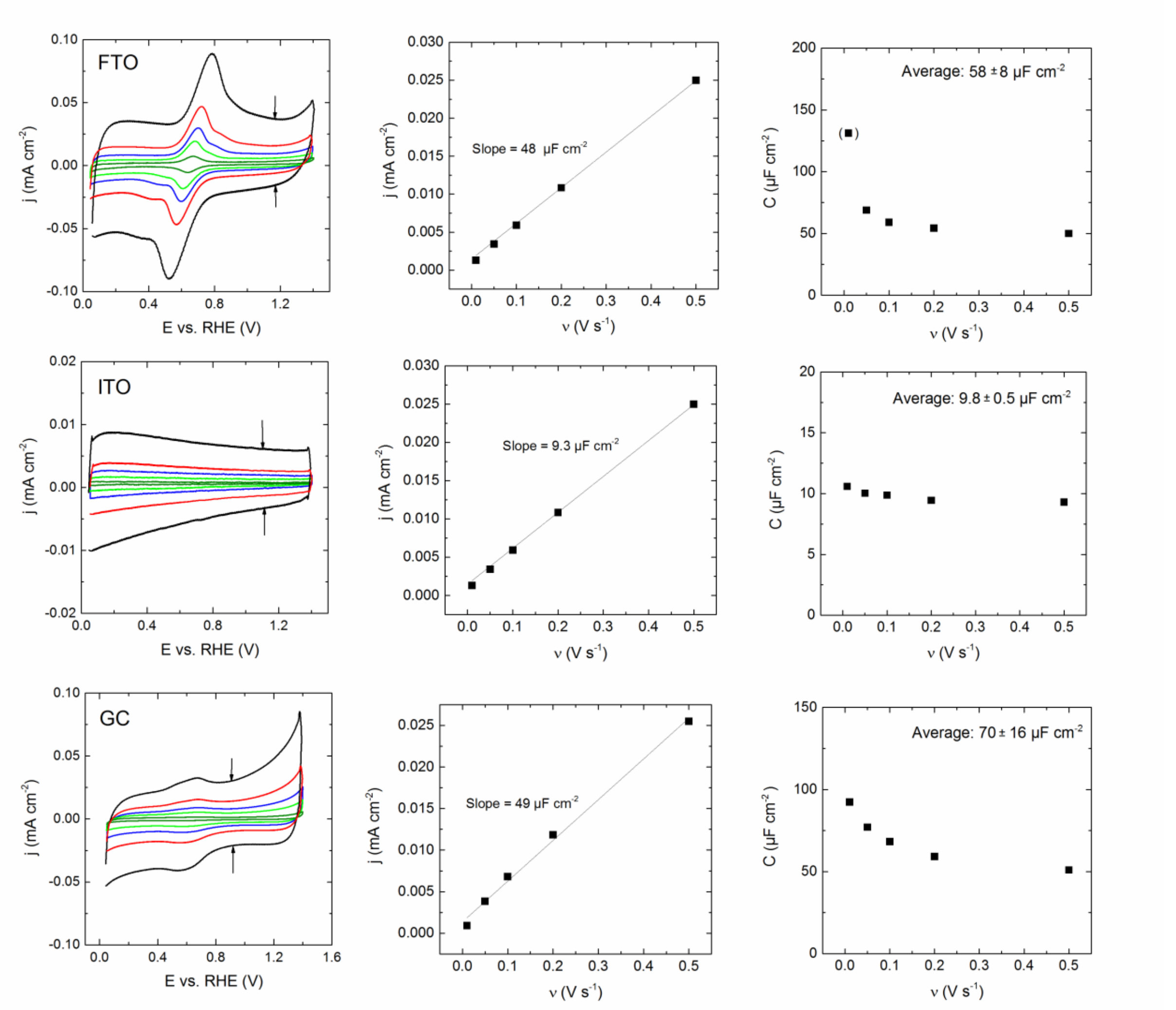

Figure S7. Estimation of the double layer capacitance on flat samples of FTO, ITO and GC using cyclic voltammetry at various scanrates (0.5, 0.2, 0.1, 0.05, 0.01 V s^{-1}).

In addition to films, powder samples were analysed using the same method. In order to measure surface area of the investigated ITO and ATO powders but not the whole electrodes, the background current originating from the GC was always subtracted. Due to the five times higher measured specific capacitance of GC vs. ITO and a relatively low amount of powders used in the dissolution studies, no significant differences were found in the CVs with and without ITO (Fig. S2). Therefore a higher loading was used for which a clear difference was observed. The ECSA was calculated to be 0.11 cm² which is one order of magnitude lower than the value provided by the supplier. According to supplier, the latter was obtained using the Brunauer–Emmett–Teller (BET) method. Assuming this ratio holds also for the lower loading, the surface area of the electrodes used in the dissolution studies was estimated as 0.0091 cm².

Unlike for ITO, specific capacitance of flat ATO samples cannot be measured. In this study it is assumed to be around 50 µF cm^{-2} as for the FTO. Even for the low loading used in the dissolution studies a significant difference in the CVs with and without ATO powder was found (Fig. S3), which justifies our assumption. The resulting ECSA value was 0.032 cm² which is again around one order of magnitude lower than the provided BET surface area.

Note the real surface area estimation of conductive oxides remains challenging especially as faradaic currents cannot be avoided. This approach does not intend to give a precise value of the ECSA, however it shows that in this case usage of the BET-surface would lead to significant overestimation of the electrochemical surface area.

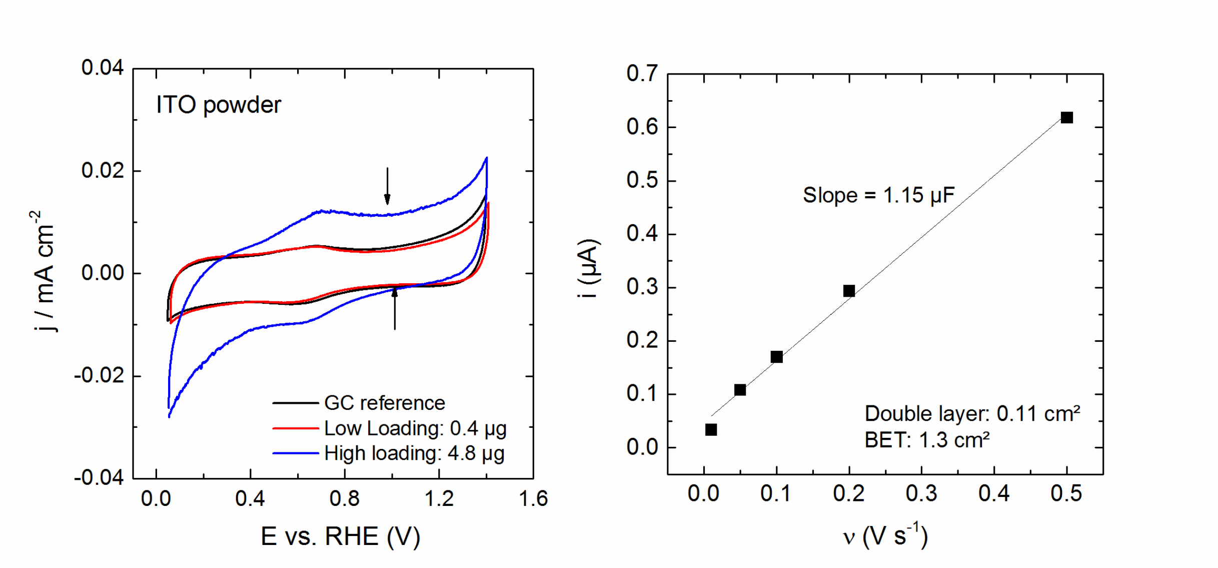

Figure S8. Estimation of the ECSA of ITO powder. Left: Cyclic voltammograms at 0.05 V s^{-1} at different loadings. Right: Background corrected current plotted vs. scanrate for the high loading of ITO.

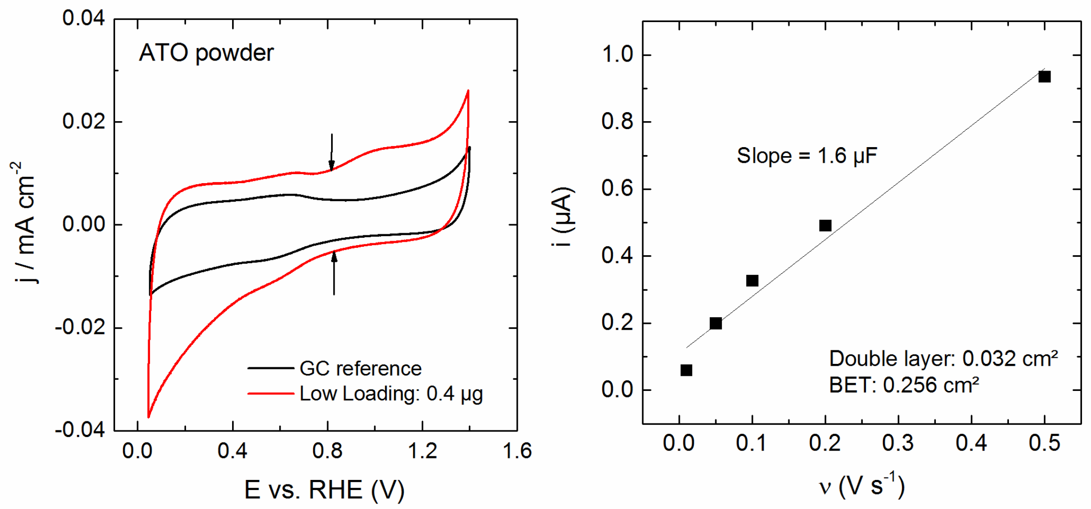

Figure S9. Estimation of the ECSA of ATO powder. Left: Cyclic voltammograms at 0.05 V s^{-1}. Right: Background corrected current plotted vs. scanrate.

References

1 Trasatti, S. & Petrii, O. A. Real surface area measurements in electrochemistry. *J. Electroanal. Chem.* **327**, 353-376 (1992).

2 McCrory, C. C. *et al.* Benchmarking hydrogen evolving reaction and oxygen evolving reaction electrocatalysts for solar water splitting devices. *J Am Chem Soc* **137**, 4347-4357, doi:10.1021/ja510442p (2015).
